# IFI44L is a novel tumor suppressor in human hepatocellular carcinoma affecting cancer stemness, metastasis, and drug resistance via regulating met/Src signaling pathway

**DOI:** 10.1186/s12885-018-4529-9

**Published:** 2018-05-30

**Authors:** Wei-Chieh Huang, Shiao-Lin Tung, Yao-Li Chen, Po-Ming Chen, Pei-Yi Chu

**Affiliations:** 1 Graduate Institute of Integrated Medicine, China Medical University, Taichung, Taiwan; 2 Department of Hematology and Oncology, Ton-Yen General Hospital, Hsinchu, Taiwan; 3 School of Medicine, Kaohsiung Medical University, Kaohsiung, Taiwan; 4 Department of General Surgery, Changhua Christian Hospital, Changhua, Taiwan; 5 Taiwan Agricultural Chemicals and Toxic Substances Research Institute, Council of Agriculture, Taichung, Taiwan; 6 School of Medicine, College of Medicine, Fu Jen Catholic University, New Taipei, Taiwan; 7 Department of Pathology, Show Chwan Memorial Hospital, No.542, Sec.1, Chung-Shang Road, Changhua City, Changhua County 50008 Taiwan, Republic of China; 8 National Institute of Cancer Research, National Health Research Institutes, Tainan, Taiwan

**Keywords:** IFI44L, Cancer stem cells, Hepatocellular carcinoma

## Abstract

**Background:**

Hepatocellular carcinoma (HCC) is the second leading cause of cancer-related death worldwide. The disease recurrent rate is relatively high resulted in poor 5-year survival in advanced HCC. Cancer stem cells (CSCs) have been considered to be one of the main mechanisms for chemoresistance, metastasis, and recurrent disease. Interferon-induced protein 44-like (IFI44L) gene is a type I interferon-stimulated gene (ISG) and belongs to the IFI44 family. Previous reports indicated antiviral activity against HCV in IFI44L, however, its precise role and function in HCC has not been unveiled.

**Methods:**

To explore the characteristics of hepatic CSCs, we successfully enriched hepatic cancer stem-like cells from three established liver cancer cell lines (Hep3B, HepG2, and PLC lines). Parental Hep3B and HepG2 cells and their sphere cells were treated with doxorubicin for 48 h and cell viability was measured by MTT assay. HCC tissue blocks from 217 patients were sampled for tissue microarray (TMA). Follow-up information and histopathological and clinical data including age, gender, tumor grade, advanced stages, HBV, HCV, tumor number, tumor size, relapse-free survival, and overall survival were obtained from the cancer registry and medical charts. The liver TMA was evaluated for IFI44L expression using immunohistochemical staining and scores.

**Results:**

These hepatic cancer stem-like cells possess important cancer stemness characteristics including sphere-forming abilities, expressing important HCC cancer stem cell markers, and more chemoresistant. Interestingly, we found that overexpression of IFI44L decreased chemoresistance towards doxorubicin and knockdown of IFI44L restored chemoresistance as well as promoted sphere formation. Furthermore, we found that depletion of IFI44L enhanced migration, invasion, and pulmonary metastasis through activating Met/Src signaling pathway. Clinically, the expression level of IFI44L significantly reduced in HCC tumor tissues. Low expression of IFI44L levels also correlated with larger tumor size, disease relapse, advanced stages, and poor clinical survival in HCC patients.

**Conclusion:**

Taken together, we first demonstrated that IFI44L is a novel tumor suppressor to affect cancer stemness, metastasis, and drug resistance via regulating Met/Src signaling pathway in HCC and can be serve as an important prognostic marker.

**Electronic supplementary material:**

The online version of this article (10.1186/s12885-018-4529-9) contains supplementary material, which is available to authorized users.

## Background

Liver cancer is the fifth most common cancer worldwide and the second leading cause of cancer-related death worldwide [1]. In primary liver cancers, most (70 to 90%) cancers are hepatocellular carcinoma (HCC) [1]. The treatment efficacy of HCC is rather low mainly due to chemoresistance and metastasis which resulted in poor 5-year survival of less than 5% in advanced HCC [2]. Cancer stem cells (CSCs) are considered to be one of the main mechanisms of chemoresistance and metastasis [3–5]. Hepatic CSCs have been identified and isolated from HCC in previous reports [2, 6–8]. To elucidate potential targetable molecular markers as well as signaling pathways of hepatic CSCs will be helpful in improving treatment efficacy in HCC.

Hepatitis B virus (HBV) or Hepatitis C virus (HCV) are hepatotropic, noncytopathic DNA viruses that cause acute and chronic necroinflammatory liver diseases and hepatocellular carcinoma [9]. Type I interferons (IFNs) are pro-inflammatory cytokines that activate JAK-STAT signaling pathways leading to transcription of IFN-stimulated genes (ISGs) to protect cells against invading viral pathogens including HBV and HCV [10–14]. Although hundreds of ISGs have been identified for the past decades, only a few have been characterized with antiviral activity. Using an overexpression screening approach, 380 human ISGs including interferon-induced protein 44-like (IF144L) gene were tested for their abilities to suppress the replication of viruses [15].

IFI44L is a type I ISG and belongs to the IFI44 family [16]. The IFI44L protein is 452 amino acid long, an approximately 47 kDa protein, and located on chromosome 1 at area p31 (GenBank AB000115). Increased expression of IFI44L was reported after treatment with IL-28A and IFN-α to inhibit HCV replication [12]. In addition, the functions of miR-9 in some cancers are recently implicated in regulating proliferation, invasion, metastasis, epithelial–mesenchymal transition (EMT), apoptosis, and tumor angiogenesis [17–19]. A previous study reported that overexpression of miR-9 significantly upregulated the expression of a lot of ISGs including IFI44L in nasopharyngeal carcinoma cells [20]. These studies indicated the promising role of IFI44L not only in anti-viral aspects but also in cancer treatment.

An earlier report documented that a novel ISG, BATF2, as potent negative regulator of hepatocyte growth factor (HGF)/Met signaling in colorectal cancer and may serve as a prognostic tumor marker [21]. IFN-α activates STAT signaling and downregulates Met in primary human hepatocytes was also reported [22]. Blocking the HGF/Met pathway by Met inhibitors or monoclonal antibodies strongly inhibits tumor growth and tumorigenicity in many malignancies including HCC [23]. Met has been known that is an upstream regulator of multiple pathways, including PI3K/Akt, Ras/MAPK, Src/Stat3, and NF-κB [22]. In liver cancer, many studies have demonstrated that Met overexpression is associated with the development of distant metastases and a shorter metastasis-free survival [23]. Consequently, Met activation is considered to be crucial for the acquisition of metastatic potential and the correlation between Met pathway and ISGs warrants further study.

In this study, we successfully enriched hepatic cancer stem-like cells and first identified that overexpression of IFI44L significantly reduces the chemoresistance towards doxorubicin and knockdown of IFI44L promotes sphere formation in HCC cells. Furthermore, we found that depletion of IFI44L expression promotes migration, invasion, and pulmonary metastasis in HCC cells. We first demonstrated that suppression of IFI44L leads to activation of Met/Src pathway. We also first identified that the expression of IFI44L decreased in tumor tissues and correlated with several poor clinical outcomes in HCC patients. Our data demonstrated that IFI44L is a potent negative regulator of Met/Src signaling pathway in modulating HCC cancer stemness and drug resistance and may serve as an important prognostic marker.

## Methods

### Patients

217 HCC tissue microarray slides were obtained from HCC patients receiving surgeries in Changhua Christian Hospital from July 2011 to November 2013 [24]. Paraffin-embedded HCC samples were obtained from Changhua Christian Hospital under the approved Institutional Review Board (IRB) protocol. Clinical patterns and overall survival data were analyzed by SPSS software and chart review. The age of all patients was between twenty-nine and eighty-one years. The clinical characteristics of these 217 patients are shown in Table 1.

**Table 1:** Relationship between clinical parameters and IFI44L expression in hepatocellular patients

		IFI44L		
Variables	*N*	Low	High	*p*-value
Age (years)				
< 65	100	49 (49%)	51 (51%)	0.892
≧65	117	56 (48%)	61 (52%)	
Gender				
Female	58	35 (60%)	23 (40%)	0.306
Male	159	75 (47%)	84 (53%)	
Differentiation				
Well	12	3 (25%)	9 (75%)	0.892
Moderate	105	55 (52%)	50 (48%)	
Poor	94	47 (50%)	47 (50%)	
Undifferentiation	6	1 (17%)	5 (83%)	
Stage				
I, II	180	84 (47%)	96 (53%)	0.029
III, IV	37	25 (68%)	12 (32%)	
Hepatitis B surface antigen				
Negative	106	52 (49%)	54 (51%)	0.892
Positive	111	56 (51%)	55 (49%)	
Hepatits C virus				
Negative	150	74 (49%)	76 (51%)	0.883
Positive	67	34 (51%)	33 (49%)	
Tumor Number				
Single	177	87 (49%)	90 (51%)	0.841
Multiple	40	21 (53%)	19 (47%)	
Tumor size				
< 5 cm	140	59 (42%)	81 (58%)	0.002
≧5 cm	77	49 (64%)	28 (36%)	
Relapse				
-	196	91 (46%)	105 (54%)	0.002
+	21	17 (81%)	4 (19%)	

### Cell culture

The human liver cancer cell lines Hep3B (ATCC number: HB-8064), HepG2 (ATCC number: HB-8065) and PLC (ATCC number: HB-8024) were obtained from the American Type Culture Collection (ATCC, Manassas, VA). All cells were cultured at 37 °C under 5% CO_2_ in Dulbecco’s modified Eagle medium (DMEM; Invitrogen) supplemented with 10% fetal bovine serum (FBS; Biological Industries) and 100 units/ml of penicilium and streptomycin (Life Technologies, Carlsbad, CA, USA).

### Vectors, antibodies, and reagents

For IFI44L-expressing vector, IFI44L coding sequence was amplified and cloned in pMSCV plasmid. Antibodies for western blotting and immunohistochemistry (IHC) are anti-IFI44L (Abcam), p-Met (Cell signaling, Tyr1234/1235), Met (Cell signaling), Src (Cell signaling) and p-Src (Cell signaling, Tyr416). IFI44L-specific siRNAs were purchased from MDBio, Inc. Detailed sequences for IFI44L siRNA oligonucleotides were shown in Additional file 1: Table S1. For cell sensitivity assays, HCC cells were pretreated with doxorubicin (Sigma-Aldrich) for 18 h (overnight) in serum-free culture medium.

### RNA extraction and qRT-PCR

Quantitative RT-PCR (qRT-PCR) was used for gene detection. Detailed procedure of reverse transcription reaction was described elsewhere [25]. qRT-PCR was performed on a CFX96 qPCR detection system (Bio-Rad) with a 1:10 dilution of cDNA by using KAPA SYBR FAST qPCR Kits (KAPA Biosystems). The mRNA levels were normalized to actin mRNA. The primers used for mRNA expression are listed in Additional file 1: Table S1.

#### Sphere-forming assay

Monolayer cells of three HCC cell lines (Hep3B, HepG2 and PLC cells) were cultured in a stem cell selective condition described previously to obtain spheres [5]. Spheres comprised at least five cells were calculated by visual counts according to a previous report [26].

#### Cell proliferation assay

The cell proliferation assay was measured by MTT assay (Promega, Madison, WI, USA). The assay was performed according to the manufacture’s protocol. Briefly, cells (with density around 3 X 10^3^ per well) were seeded in 96-well plates and were incubated for 24 h. Cells were subsequently treated with various concentrations of doxorubicin and then were incubated for 48 h. Viable cells with active metabolism converted MTT into a formazan product, the quantity of which was measured at a wave length of 490 nm with 96-well plate reader and was directly proportional to the number of viable cells. The drug concentration required to reduce proliferation by 50% is defined as IC_50_. All the experiments were performed in triplicates and repeated three times.

### Cell chemotatic migration and invasion assay

Migration and invasion abilities of HCC cells were carried out using the Falcon Cell Culture Inserts with or without Matrigel (BD Biosciences) coating as described previously [27]. Detailed procedures were described elsewhere [25].

### In vivo metastasis assays

Hep3B Cells (1 × 10^6^) with indicated treatments were suspended in phosphate-buffered saline (PBS) and were injected individually into the tail vein of 6- to 8-week-old C.B-17 severe-combined immunodeficient (CB17-SCID) mice. All mice were monitored meticulously and were sacrificed after 40 days of implantation. Tumor growth was observed by live animal BLI (Caliper IVIS system, PerkinElmer).

### Immunohistochemistry (IHC)

IHC was performed to detect IFI44L expression from paraffin-embedded HCC specimens. The slides were stained with anti-IFI44L antibody (Bethyl Labs, Montgomery, TX, USA) [28]. The IFI44L antibody was purchased from ThermoFisher (Rock, USA). In liver cancer specimens, the detailed scores for IHC were defined as described previously [24, 29].

### Statistical analysis

The SPSS software (Version 13.0 SPSS Inc., Chicago, IL, USA) was used to conduct Chi-square analysis and paired-samples t-test. Kaplan-Meier method was performed for analyzing survival data. Variables related to survival were analyzed using Cox’s proportional hazards regression model via SPSS software. Differences between experimental groups were calculated using the Mann–Whitney U test. Differences with *P* values of < 0.05 are considered statistically significant.

## Results

### Successful enrichment of human HCC cancer stem-like cells from Hep3b, HepG2, and PLC lines

**Fig. 1:**
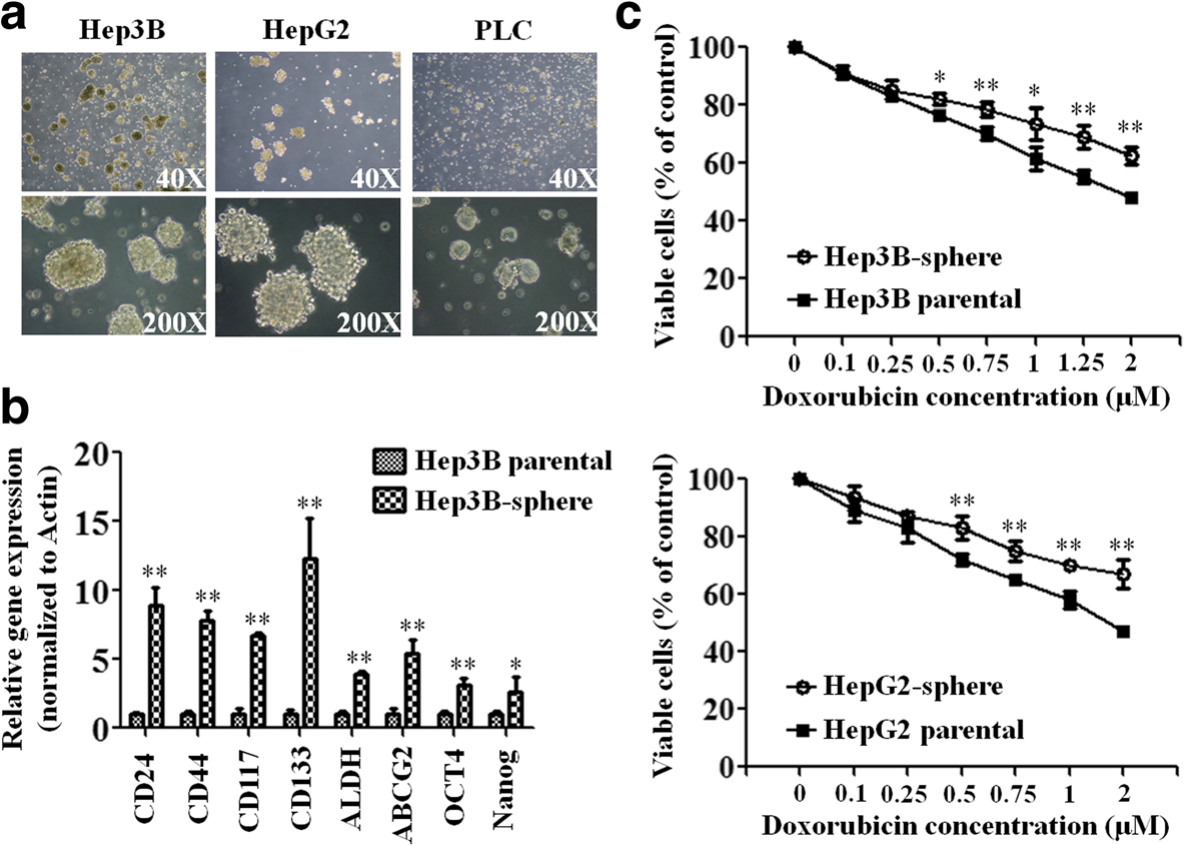
HCC cancer stem-like cells were successfully enriched from Hep3B, HepG2, and PLC cell lines. **a** Formation of spheres under the stem cell selective condition on day 8 after culturing from parental Hep3B, HepG2, and PLC cells is shown. **b** The mRNA expression levels of HCC cancer stem cell markers in parental Hep3B cells and their sphere cells were analyzed by qRT–PCR with actin as an internal control. Histograms represent means ± s.d. from three independent experiments (*, *P* < 0.05; **, *P* < 0.01). **c** Dose-dependent growth inhibition of parental Hep3B and HepG2 cells and their sphere cells upon continuous exposure to the indicated concentrations of doxorubicin for 48 h was measured by MTT assay. Each dosage point represents the mean ± s.e. from three independent experiments (*, *P* < 0.05; **, *P* < 0.01)

In order to enrich for CSCs, parental Hep3B, HepG2, and PLC cells from monolayer were cultured in a stem cell selective condition described in ‘Methods’ to form spheres. Most of the suspended cells underwent apoptosis during the first 2 days of culturing, and the rest of survived cells gradually formed floating spheres. The spheres grew larger and often reached to 50–100 μM in diameter after 4–8 days (Fig. 1a). Overexpression of mRNA of HCC cancer stem cell markers was found in.

Hep3B sphere cells compared with their parental cells. These cancer stem cell markers, including CD24, CD44, CD117, CD133, ALDH, ABCG2, OCT4, and Nanog, were significantly higher in Hep3B sphere cells shown by qRT–PCR analysis (Fig. 1b) [7, 30–37].

Next, we examined the chemosensitivity of these sphere cells. Parental Hep3B and HepG2 cells and their sphere cells were treated with doxorubicin for 48 h and cell viability was measured by MTT assay. Hep3B and HepG2 sphere cells are found to be more chemoresistant to continuous exposure to various concentrations of doxorubicin (Fig. 1c). Thus, we have successfully enriched HCC cancer stem-like cells from Hep3B, HepG2, and PLC lines displaying cancer stem cell characteristics including sphere-forming, expression of HCC cancer stem cell markers, and more chemoresistant in accordance with established parameters of cancer stem-like cells [38–40].

### Overexpression of IFI44L restores chemosensitivity and knockdown of IFI44L promotes sphere formation

**Fig. 2:**
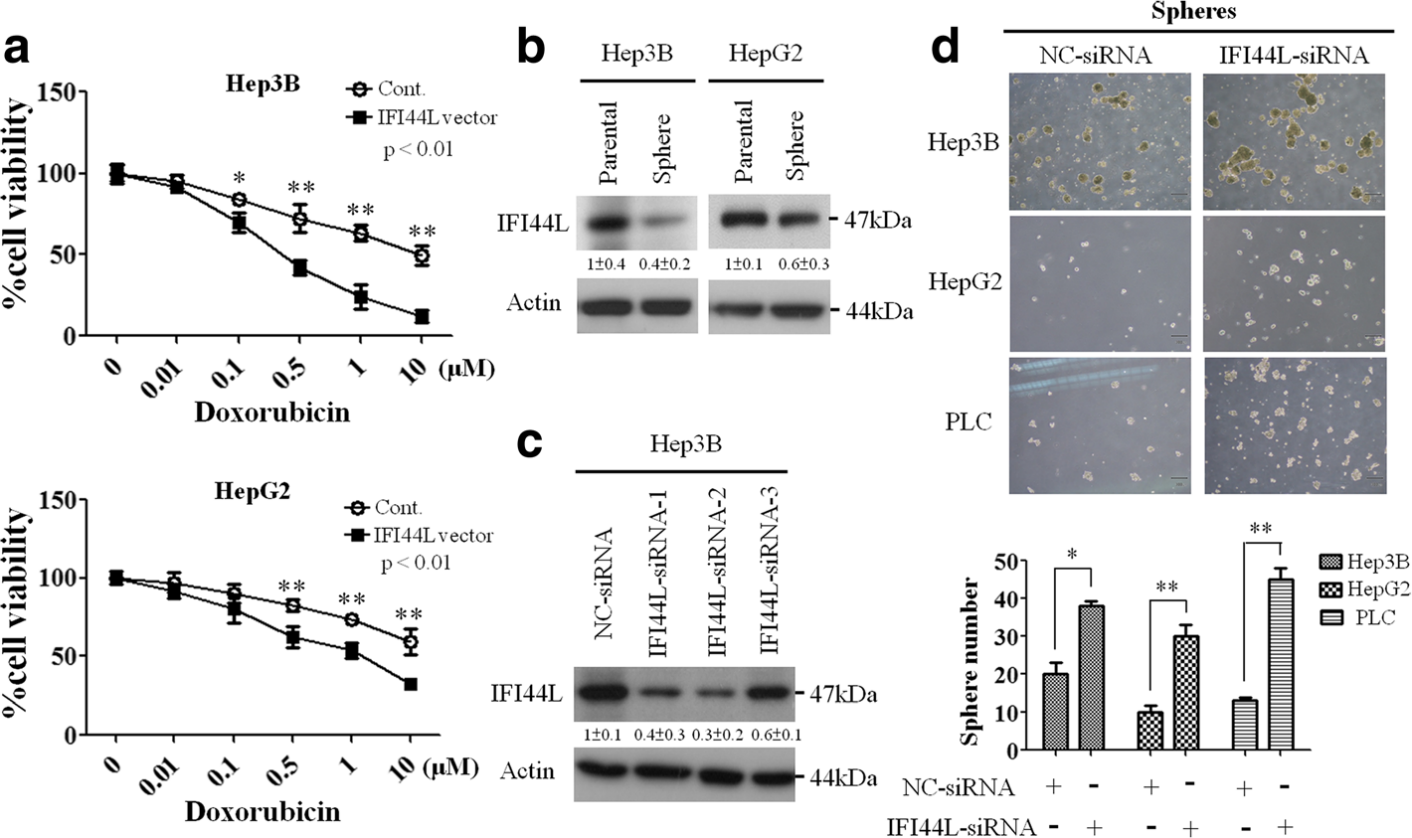
The effects of IFI44L on drug resistance and sphere formation. **a** Dose-dependent growth inhibition of Hep3B and HepG2 cells upon continuous exposure to the indicated concentrations of doxorubicin for 48 h was measured by MTT assay (*, *P* < 0.05; **, *P* < 0.01). Cells were transfected with 1 μg of pMSCV or pMSCV-IFI44L expression plasmids (IFI44L vector). **b** The expression levels of IFI44L in parental Hep3B and HepG2 cells and their sphere cells were measured by Western blotting. The actin was used as an internal control. Relative band intensity was quantified by ImageJ 1.42 (Windows version of NIH Image, http://rsb.info.nih.gov/ij/) and was represented with normalized mean ± s.e. (*n* = 3) below each band. **c** Western blotting analysis of three different siRNAs against IFI44L in Hep3B cells. The actin was used as an internal control. Relative band intensity was quantified by ImageJ 1.42 and was represented with normalized mean ± s.e. (*n* = 3) below each band. **d** Sphere formation under stem cell selective condition was examined on day 8 after culturing of the cells transfected with the indicated IFI44L-siRNA. The original magnification was 40X. Histograms represent means ± s.d. from three independent experiments (*, *P* < 0.05, **, *P* < 0.01)

Since IFI44L was implied to be correlated with cancer [20], we then investigated the impact of IFI44L on drug resistance. Cells transfected with IFI44L expression plasmid or control plasmid were tested their protein expression of IFI44L to confirm the transfection efficiency. Western blotting showed upregulation of IFI44L protein level in Hep3B and HepG2 cells after transfection with the expression plasmid of IFI44L (IFI44L vector) (Additional file 2: Fig. S1). Our data indicated that Hep3b and HepG2 cells became more chemosensitive to continuous exposure to different doses of doxorubicin after transfection with IFI44L vector (Fig. 2a), whereas IFI44L knockdown restored their chemoresistance (Additional file 3: Figure S2). These data suggested that overexpression of IFI44L significantly decreased chemoresistance of HCC lines towards doxorubicin. To assess whether IFI44L level correlated with cancer stemness in HCC, we examined the protein expression level of IFI44L in HCC lines. Decrease of IFI44L protein level in Hep3b and HepG2 sphere cells was found compared with their parental cells by Western blotting analysis (Fig. 2b). We then investigated if suppression of IFI44L by its small interfering RNAs (siRNA) could inhibit cancer stemness characteristics in HCC lines. Three specific IFI44L-siRNAs were tested for their inhibitory efficacy by analyzing the IFI44L protein levels in Hep3B, HepG2 and PLC cells, IFI44L-siRNA-2 showed the highest knockdown effect in inhibiting IFI44L protein and it was used in the subsequent experiments (Fig. 2c, Additional file 4: Figure S3). Next we tested whether sphere-forming ability of Hep3B, HepG2, and PLC lines could be promoted by knockdown of IFI44L. After 8 days culturing of Hep3b, HepG2, and PLC cells in the stem cell selective condition, sphere number was calculated by visual counting under microscope. Knockdown of IFI44L caused significant increase of sphere number (Fig. 2d). Thus, our data suggested that IFI44L may play as a tumor suppressor role in restoring chemosensitivity and affecting cancer stemness.

### Depletion of IFI44L expression promotes migration, invasion and pulmonary metastasis and implicates in met/Src signaling pathway in HCC

**Fig. 3:**
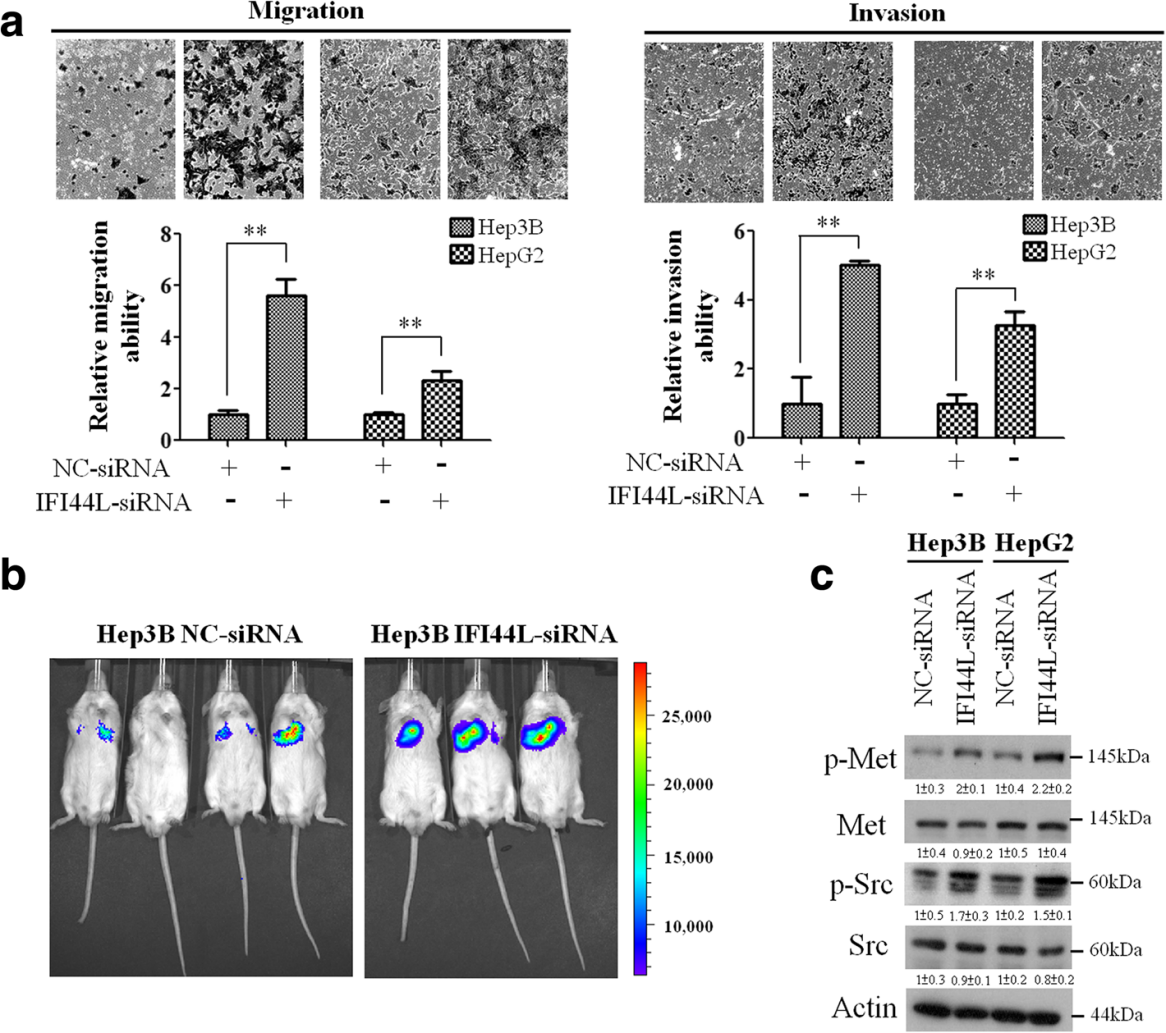
IFI44L functions as a tumor suppressor affecting metastasis implicates with Met/Src signaling. **a** Analysis of the effect of IFI44L on Hep3B and HepG2 cell migration and invasion using Boyden chamber assay. Quantitative data are shown by histograms and representative photographs of the migrated/invaded cells from different treatments are shown. **b** Representative xenograft tumors formed by 5 × 10^5^ Hep3B sphere cells in the SCID mice. Tumor growth was monitored by BLI. Representative BLIs are shown on day 30 after implantation. **c** The protein expression levels of the signaling components of the Met/Src signaling in Hep3B and HepG2 cells are shown by Western blotting. The actin was used as an internal control. Relative band intensity was quantified by ImageJ 1.42 and was represented with normalized mean ± s.e. (*n* = 3) below each band

Furthermore, we evaluate the tumor suppressor role of IFI44L in regulating cancer metastasis. In Boyden chamber assay, we found that depletion of IFI44L expression significantly promotes Hep3B, HepG2 and PLC cell migration and invasion abilities (Fig. 3a and Additional file 5: Figure S4). To investigate whether IFI44L regulated cancer cell metastasis in vivo, we employed an experimental metastasis model via tail vein injection in SCID mice. In this model, knockdown of IFI44L significantly promoted lung metastasis of Hep3B cells compared with the control group (Fig. 3b). Since ISGs are implied to be correlated with Met pathway [11, 22, 23], we then explored the role of IFI44L in Met signaling pathway. By Western blotting analysis, we found that suppression of IFI44L enhances the phosphorylation of Met and Src in Hep3B and HepG2 cells (Fig. 3c). To further assess the role of IFI44/Met/Src axis in regulating cancer metastasis, we performed additional Western blotting analysis as well as migration and invasion assay. We found that overexpression of IFI44L decreased phosphorylation of Met as well as migration and invasion abilities in Hep3B cell line, whereas ectopic expression of Met reversed IFI44L-mediated inhibition of migration and invasion abilities approximately 50% (Additional file 6: Figure S5). Taken together, these findings reinforced that the functional role of IFI44L as a tumor suppressor and it could implicate in Met/Src signaling pathway in HCC.

### The expression level of IFI44L significantly decreased in HCC tumor tissues

**Fig. 4:**
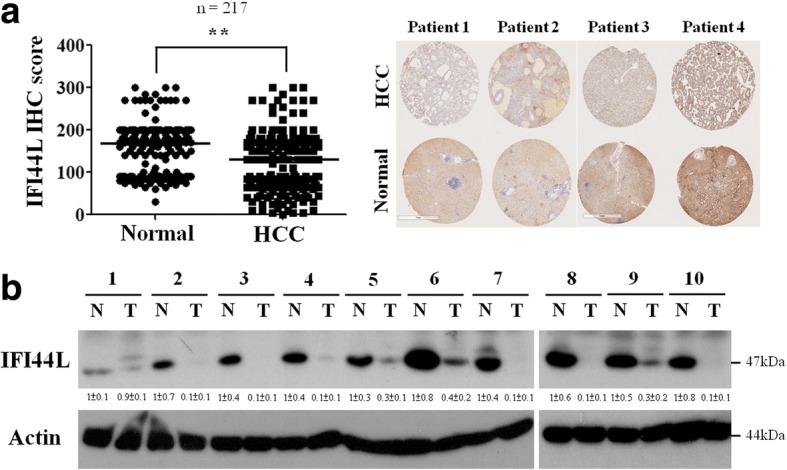
The expression level of IFI44L decreased in HCC tumor tissues. **a** The level of IFI44L was examined by IHC staining in 217 pairs of HCC tumor tissues and their adjacent normal tissues (**, *P* < 0.01). **b** Images of Western blotting analyses of IFI44L protein level in ten matched pairs of HCC tumor tissues and adjacent normal tissues. The actin was used as an internal control. Relative band intensity was quantified by ImageJ 1.42 and was represented with normalized mean ± s.e. (*n* = 3) below each band

To evaluate the correlation of IFI44L with clinical samples, the expression of IFI44L in 217 pairs of normal liver and HCC tumor tissues were analyzed by IHC and Western blotting analysis. The IHC score of IFI44L was significantly higher in normal liver tissues compared with tumor tissues (Fig. 4a). Western blotting analysis also revealed that all of ten pairs of matched HCC tumor tissues expressed lower level of IFI44L in comparison with the matched normal tissues (Fig. 4b). Downregulation of IFI44L expression found in HCC tumor tissues is compatible with the tumor suppressor role in HCC we discovered above.

### Dowregulation of IFI44L expression levels significantly correlated with larger tumor size, disease relapse, advanced stages, and poor clinical survival in HCC patients

**Fig. 5:**
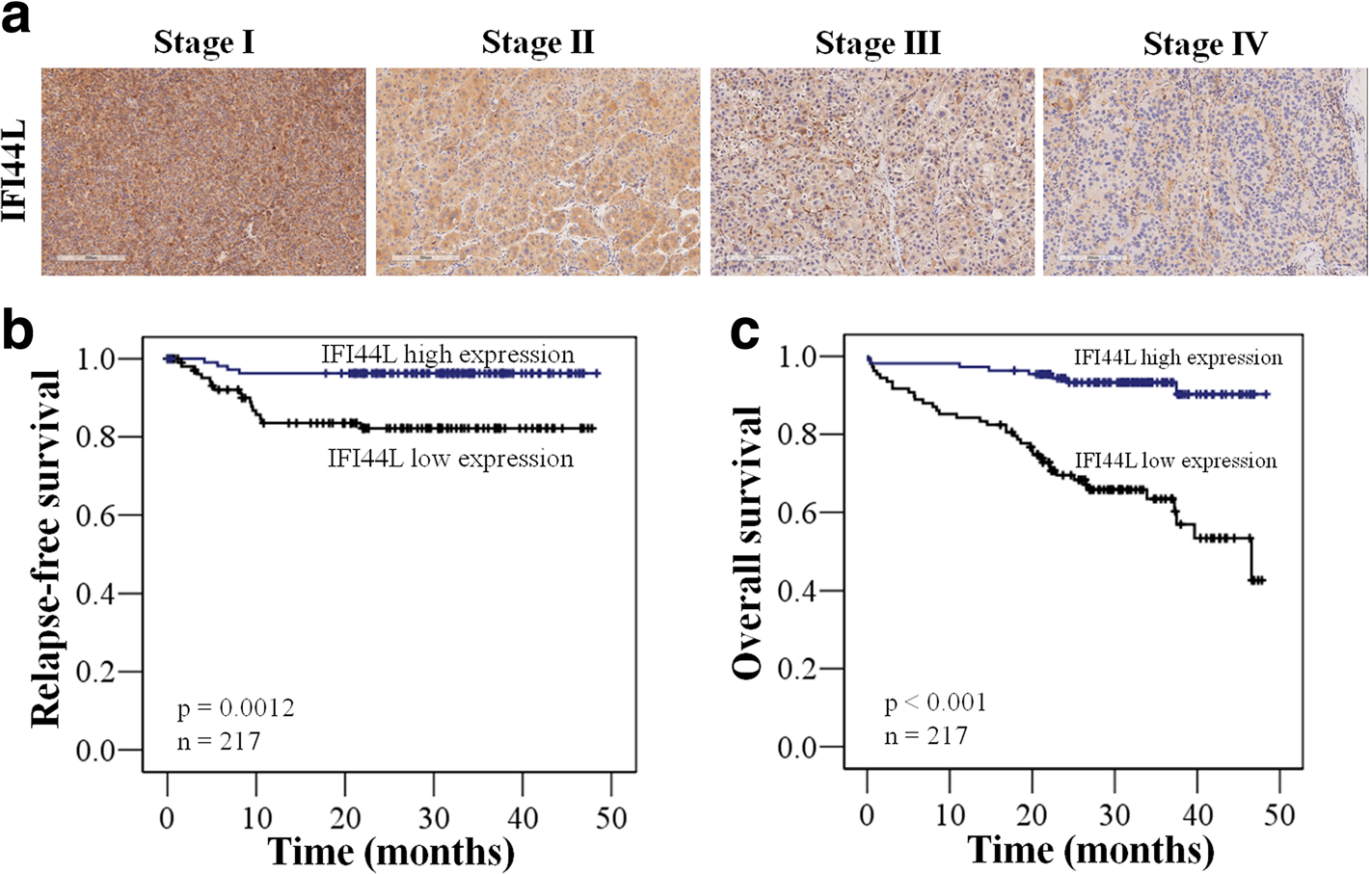
The expression level of IFI44L correlates with clinical staging, RFS, and OS in HCC patients. **a** Representative examples of the expression levels of IFI44L protein determined by IHC of clinical specimens. **b** The mRNA expression level of IFI44L correlates with RFS in 217HCC patients. **c** The mRNA expression level of IFI44L correlates with OS in 217 HCC patients

Furthermore, the correlation between clinicopathological characteristics and IFI44L of these 217 patients were analyzed in Table 1. Among these parameters, age, gender, tumor differentiation, HBV surface antigen, anti-HCV antibody, and the tumor number were not significantly different in patients with low versus high expression levels of IFI44L (Table 1). However, low expression of IFI44L was observed in only 47% (84/180) of the early stages (stage I/II) HCC patents whereas 68% (25/37) of the late stages (stage III/IV) HCC patients expressed low levels of IFI44L (*P* = 0.029) (Table 1). IHC staining also confirmed that IFI44L protein level decreased markedly in advanced stages in HCC samples (Fig. 5a). Moreover, higher percentage of HCC patients with low expression level of IFI44L had larger tumor size then patients with high expression level of IFI44L (64% vs 36%, *P* = 0.002) (Table 1). Since CSCs are indicated to be associated with cancer recurrence [2, 38], our previous experiments also indicated that IFI44L affects cancer stemness in HCC cells. In clinic data, we also found that patients with low expression level of IFI44L had significantly higher relapse rate (81% vs 19%, *P* = 0.002) and shorter relapse-free survival (RFS) (*p* = 0.0012) than patients with high expression level of IFI44L (Table 1, Fig. 5b).

In survival analysis, the influence of clinicopathological characteristics including IFI44L on patients’ overall survival (OS) was statistically examined by univariate analysis shown in Table 2. Four parameters including advanced stages, larger tumor size, disease relapse, and low expression of IFI44L are significant correlated with shorter median OS (*P* < 0.001) (Table 2). Kaplan–Meier survival analysis of these 217 patients also revealed that low expression level of IFI44L correlated with poor OS (*P* < 0.001) (Fig. 5c). These results suggested that downregulation of IFI44L expression levels significantly correlated with larger tumor size, disease relapse, advanced stages, and poor clinical survival in HCC patients and could serve as an important prognostic marker.

**Table 2:** Univariate analysis of influence of clinical characteristics on overall survival in hepatocellular patients

		OS		
Characteristics	*N*	Median survival (months)	Survival (%)	Log-rank
Age (years)				
< 65	100	36.83	75.00%	0.282
≧65	117	41.42	80.34%	
Gender				
Female	58	38.37	76.27%	0.631
Male	159	40.49	78.48%	
Differentiation				
Well, Moderate	117	40.69	78.45%	0.731
Undifferentitation, Poor	100	39.26	77.23%	
Stage				
I, II	180	42.22	82.49%	< 0.001
III, IV	37	30.13	57.50%	
Hepatitis B surface antigen				
Negative	106	40.33	79.25%	0.617
Positive	111	39.08	76.58%	
Hepatits C virus				
Negative	150	39.14	76.67%	0.489
Positive	67	41.1	80.60%	
Tumor Number				
Single	177	40.25	77.78%	0.926
Multiple	40	37.17	78.38%	
Tumor size				
< 5 cm	140	49.79	86.43%	< 0.001
≧5 cm	77	33.12	62.34%	
Relapse				
-	196	41.31	81.54%	< 0.001
+	21	28.79	45.00%	

## Discussion

HCC has been a global health problem with rising incidence in Western countries recently [1]. In the West, around 40% of patients are diagnosed as early Barcelona Clinic Liver Cancer (BCLC) stages and are eligible for potential curative treatment such as surgical resection, radiofrequency ablation, microwave ablation, percutaneous alcohol injection, and liver transplantation [9, 41–43]. However, the probability of disease recurrence is around 50% within 3 years after successful treatment [2]. Hepatic CSCs exhibit multidrug and radio-resistant properties and are considered as in part the main mechanism of chemoresistance and recurrent disease [2, 4]. In our study, we successfully enriched cancer stem-like cells via sphere-forming method in nonadhesive culture plates with serum-free culture medium from three hepatic cancer cell lines. These cancer stem-like cells express important hepatic CSC markers such as CD24, CD44, CD117, CD133, ALDH, ABCG2, Oct4, and Nanog which were extensively reported before [7, 30–37]. They also reveal significant chemoresistance towards doxorubicin in accordance with previous reports [2]. To find specific molecules to target these cancer stem-like cells would be very important in treating HCC.

Type I IFNs are a family of cytokines to directly activate the transcription of ISGs to exert anti-viral, anti-proliferative, and immunomodulatory activities [10, 11]. IFI44L, one of the type I ISG, exhibits a low antiviral activity against HCV and is indicated to be correlated with some cancer recently although the reports are scarce [12, 20, 44]. In present study, our data showed that overexpression of IFI44L restores chemosensitivity towards doxorubicin whereas decreased expression of IFI44L promotes sphere formation in HCC cell lines. Depletion of IFI44L also enhanced migration, invasion, and lung metastasis in HCC cells. According to the above results, IFI44L was proposed as a novel tumor suppressor modulating cancer stemness, drug resistance, migration and invasion, as well as pulmonary metastasis in HCC. Although one recent study indicated that upregulation of IFI44L was significantly correlated with shorter overall survival and shorter median survival time in pancreatic ductal adenocarcinoma [44], our data revealed that low expression of IFI44L was found in HCC tumor samples and was correlated with larger tumor size, more disease relapse, advanced stages as well as significant poorer RFS and OS. Although some study identified that IFI44L overexpressed in pancreatic ductal adenocarcinoma and correlated with worse clinical prognosis, this conclusion is only made from statistics of databases collecting from gene expression profiling and TCGA database but lacks in vitro and in vivo experimental confirmation [44]*.* The functional role of IFI44L in different cancers still warrants further study.

In advanced stages of HCC, conventional chemotherapy such as doxorubicin, cisplatin, and 5-fluorouracil were generally introduced but the response rate was very low (from 15 to 20%) and these chemotherapeutic agents failed to prolong survival [2, 41]. Sorefenib, a small molecule multikinase inhibitor that inhibits tumor-cell proliferation and tumor angiogenesis, is the first targeted therapy to reveal survival benefit in patients with advanced HCC [41]. Other new molecular pathways including.

Ras/Raf/MEK/ERK (MAPK) pathway, wnt/catenin pathway, PI3K/Akt/mTOR pathway, VEGF pathway, and HGF/Met pathway etc. were extensively explored in HCC patients [9, 23, 45]. The efficacy of new targeted therapies such as lenvatinib, nivolumab, ramucirumab, tivantinib, and cabozantinib etc. are still under evaluation in large clinical trials [9]. Of the above mentioned pathways, the HGF/Met pathway has been implicated in tumor cell migration, invasion, proliferation, and angiogenesis [23]. High expression of Met and HGF was reported to be correlated with early recurrence of HCC after hepatectomy and shorter survival in HCC patients [23]. Several studies indicated that IFN regulates multiple STAT signaling and downregulates Met resulting in suppression of HGF-induced signals and cell proliferation [14, 22]. In our study, we first identified that suppression of IFI44L leads to the activation of Met/Src pathway. Thus, the phenomenon that suppression of IFI44L promotes cancer stemness, migration, invasion, and pulmonary metastasis in HCC cells and overexpression of IFI44L results in restoring chemosensitivity observed in our study might be regulated via affecting Met/Src signaling pathway.

## Conclusion

Our study has demonstrated that IFI44L as a novel tumor suppressor in HCC through perturbation of Met/Src signaling. Clinical relevance of low expression of IFI44L with larger tumor size, disease relapse, advanced stages, and poor outcomes in HCC patients was also first identified. The IFI44L could serve as a prognostic biomarker and a promising therapeutic target in the treatment of HCC.

## Additional files

Additional file 1: **Table S1.** siRNA sequences and qRT-PCR primers used in this study. (TIF 523 kb)

Additional file 2: **Figure S1.** The protein expression levels as reflected by Western blotting of IFI44L in Hep3B and HepG2 cells transfected with the IFI44L expression vector are shown. The actin was used as an internal control. Relative band intensity was quantified by ImageJ 1.42 (Windows version of NIH Image,http://rsb.info.nih.gov/ij/) and was represented with normalized mean ± s.e. (*n* = 3) below each band. (TIF 105 kb)

Additional file 3: **Figure S2.** Dose-dependent growth inhibition of Hep3B and HepG2 cells upon continuous exposure to the indicated concentrations of doxorubicin for 48 h was measured by MTT assay. Cells were transfected with 20 nM of control (NC-siRNA) or IFI44L-siRNA (*, *P* < 0.05; **, *P* < 0.01). (TIF 160 kb)

Additional file 4: **Figure S3.** Western blotting analysis of three different siRNAs against IFI44L in HepG2 and PLC cells. The actin was used as an internal control. Relative band intensity was quantified by ImageJ 1.42 and was represented with normalized mean ± s.e. (*n* = 3) below each band. (TIF 168 kb)

Additional file 5: **Figure S4.** Analysis of the effect of IFI44L on PLC cell migration and invasion using Boyden chamber assay. Quantitative data are shown by histograms and representative photographs of the migrated/invaded cells from different treatments are shown. Histograms represent means ± s.d. from 3 independent experiments (**, *P* < 0.01). (TIF 252 kb)

Additional file 6: **Figure S5.** Ectopic expression of Met significantly restored IFI44L expression-mediated inhibition of migration and invasion abilities. Left, overexpression of IFI44L reduced the phosphorylation of Met, which could be partially rescued by transfecting Met vector. The actin was used as an internal control. Relative band intensity was quantified by ImageJ 1.42 and was represented with normalized mean ± s.e. (*n* = 3) below each band. Right, the migration and invasion abilities affected by overexpression of IFI44L and ectopic expression of Met in Hep3B cell line. Quantitative data are shown by histograms and representative photographs of the migrated/invaded cells from different treatments are shown. Histograms represent means ± s.d. from 3 independent experiments (*, *P* < 0.05; **, *P* < 0.01). (TIF 437 kb)

## Abbreviations

CSCs: Cancer stem cells
EMT: Epithelial–mesenchymal transition
HBV: Hepatitis B virus
HCC: Hepatocellular carcinoma
HCV: Hepatitis C virus
HGF: Hepatocyte growth factor
IF144L: Interferon-induced protein 44-like
IFNs: Type I interferons
ISG: Type I interferon-stimulated gene
siRNA: small interfering RNAs
