# Supplementary figures and images for: IFI44L is a novel tumor suppressor in human hepatocellular carcinoma affecting cancer stemness, metastasis, and drug resistance via regulating met/Src signaling pathway
Source: BMC Cancer. 2018 May 30;18:609. doi: 10.1186/s12885-018-4529-9 (PMC5977745; doi:10.1186/s12885-018-4529-9)

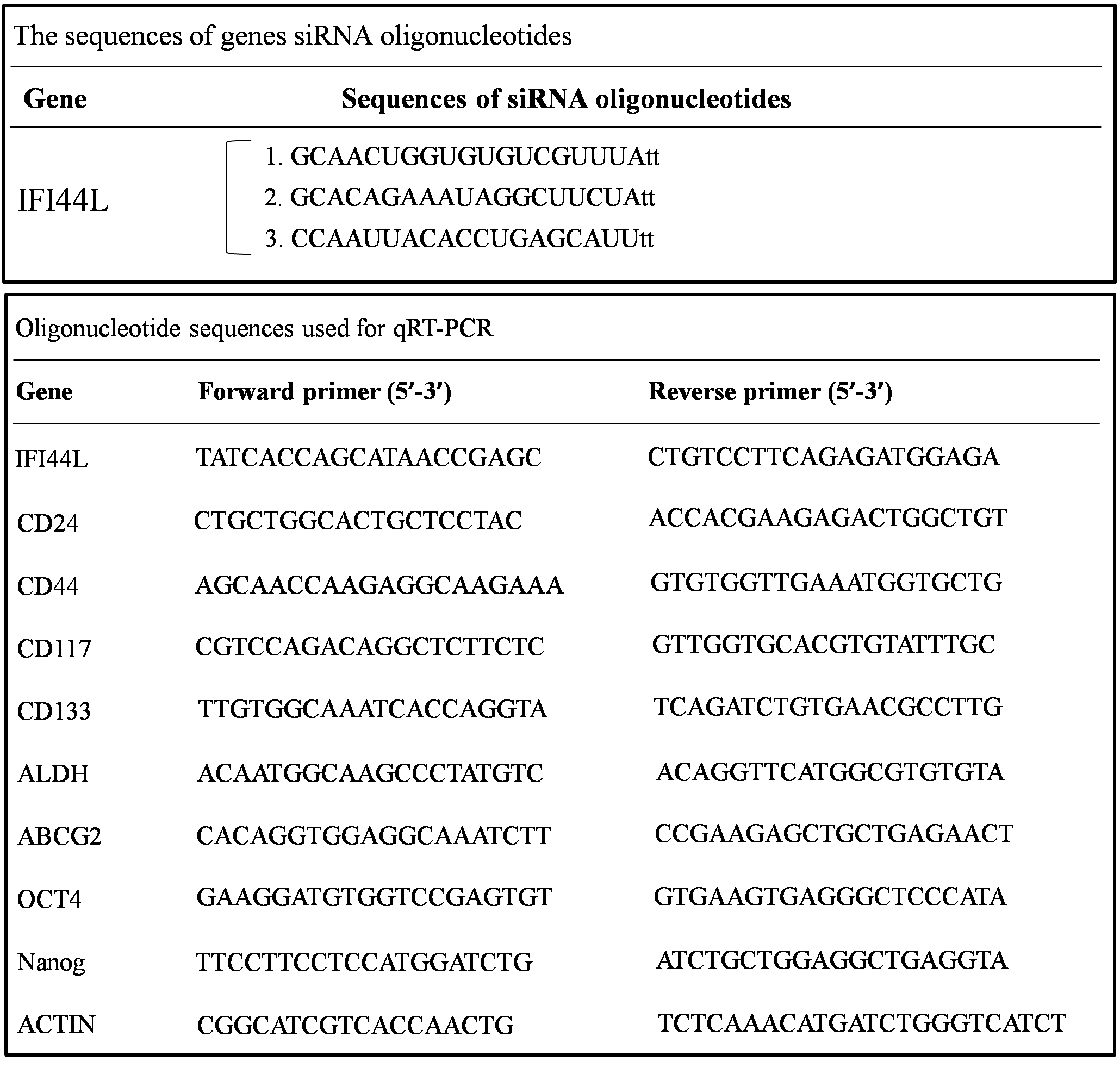

Supplement: Supplementary file 1 — Table S1. siRNA sequences and qRT-PCR primers used in this study. (TIF 523 kb) [file 12885_2018_4529_MOESM1_ESM.tif]

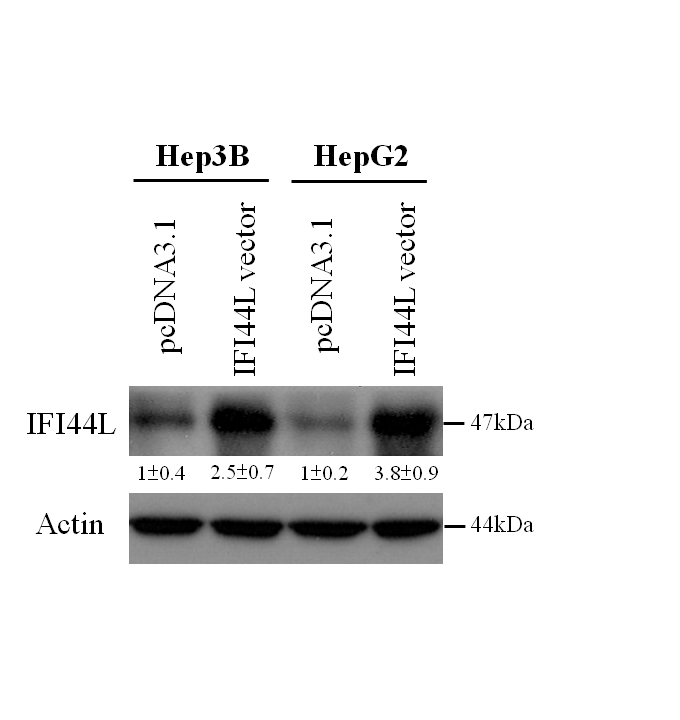

Supplement: Supplementary file 2 — Figure S1. The protein expression levels as reflected by Western blotting of IFI44L in Hep3B and HepG2 cells transfected with the IFI44L expression vector are shown. The actin was used as an internal control. Relative band intensity was quantified by ImageJ 1.42 (Windows version of NIH Image,http://rsb.info.nih.gov/ij/) and was represented with normalized mean ± s.e. (n = 3) below each band. (TIF 105 kb) [file 12885_2018_4529_MOESM2_ESM.tif]

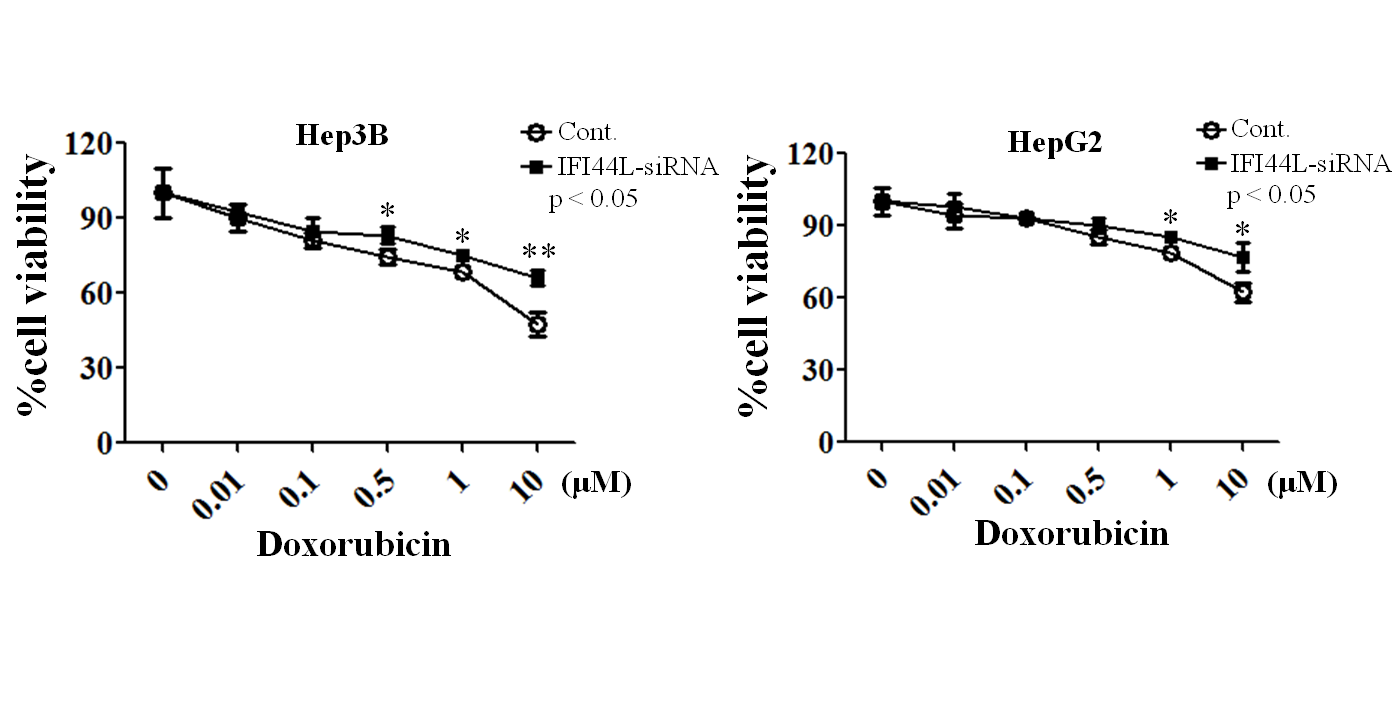

Supplement: Supplementary file 3 — Figure S2. Dose-dependent growth inhibition of Hep3B and HepG2 cells upon continuous exposure to the indicated concentrations of doxorubicin for 48 h was measured by MTT assay. Cells were transfected with 20 nM of control (NC-siRNA) or IFI44L-siRNA (*, P < 0.05; **, P < 0.01). (TIF 160 kb) [file 12885_2018_4529_MOESM3_ESM.tif]

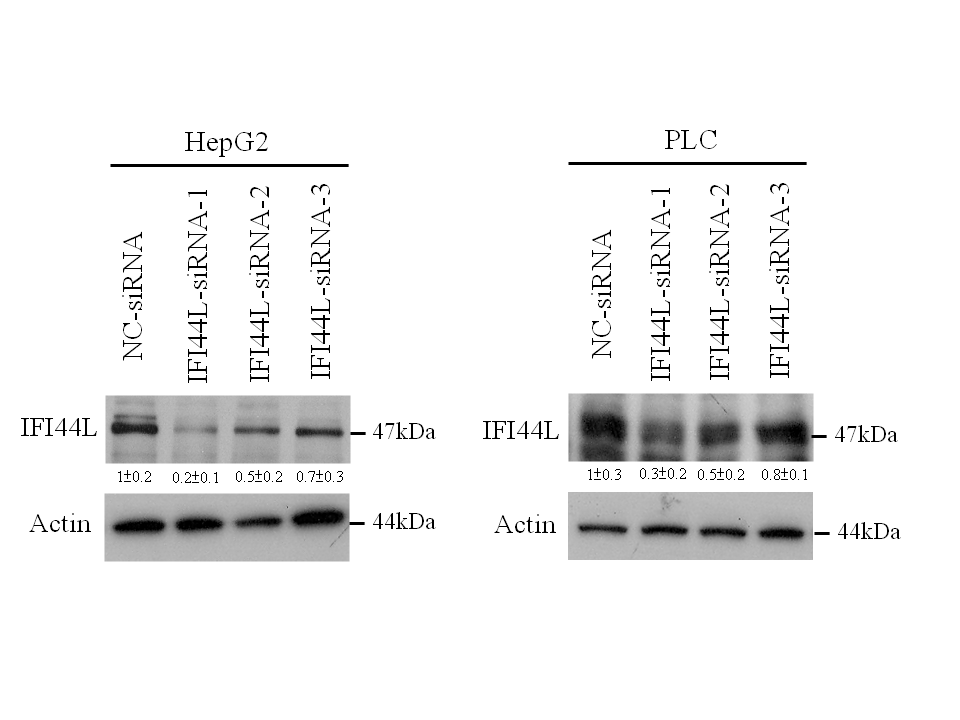

Supplement: Supplementary file 4 — Figure S3. Western blotting analysis of three different siRNAs against IFI44L in HepG2 and PLC cells. The actin was used as an internal control. Relative band intensity was quantified by ImageJ 1.42 and was represented with normalized mean ± s.e. (n = 3) below each band. (TIF 168 kb) [file 12885_2018_4529_MOESM4_ESM.tif]

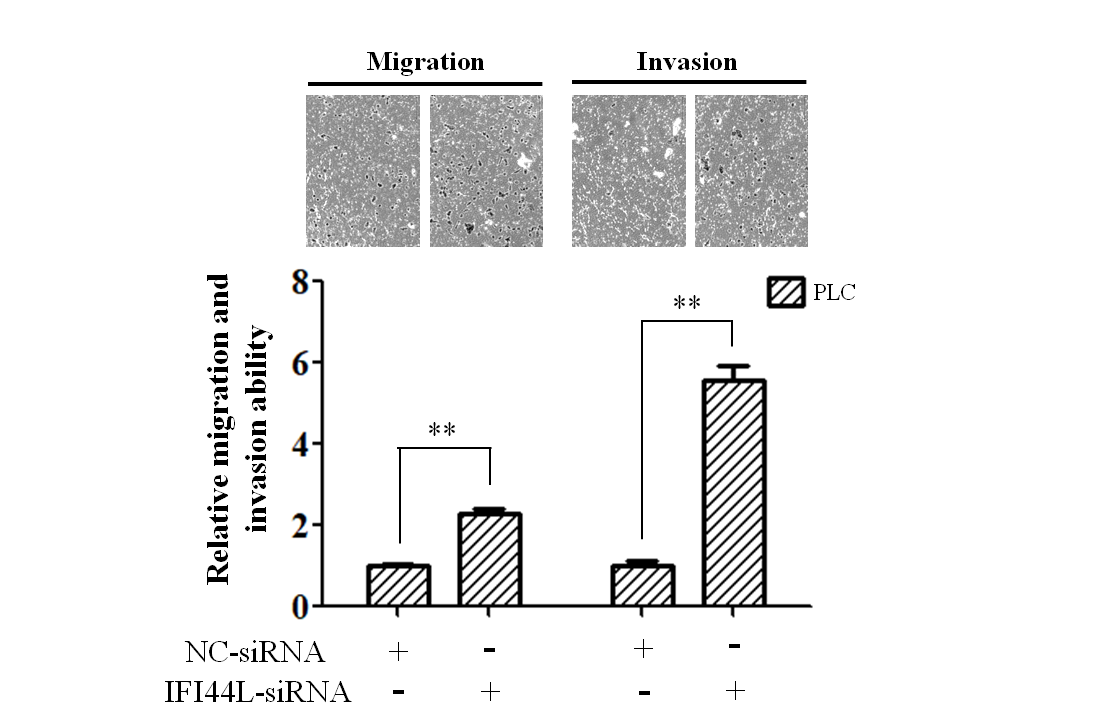

Supplement: Supplementary file 5 — Figure S4. Analysis of the effect of IFI44L on PLC cell migration and invasion using Boyden chamber assay. Quantitative data are shown by histograms and representative photographs of the migrated/invaded cells from different treatments are shown. Histograms represent means ± s.d. from 3 independent experiments (**, P < 0.01). (TIF 252 kb) [file 12885_2018_4529_MOESM5_ESM.tif]

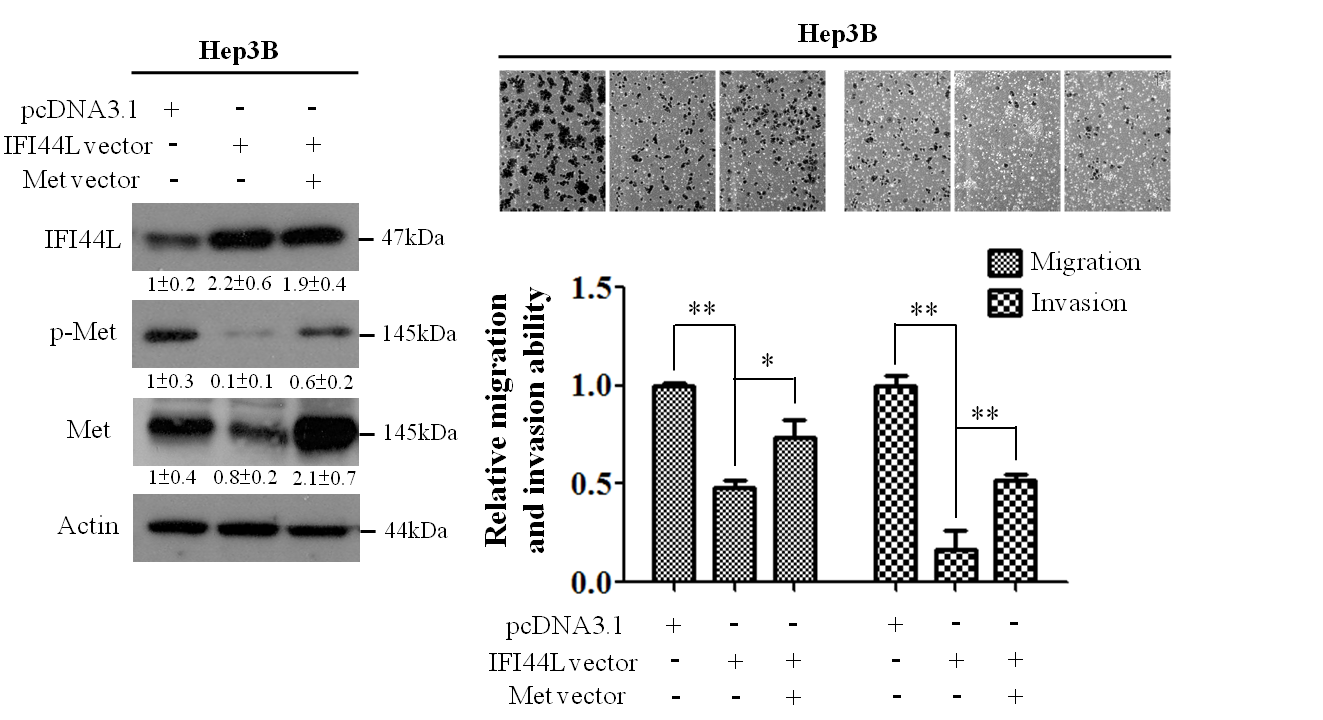

Supplement: Supplementary file 6 — Figure S5. Ectopic expression of Met significantly restored IFI44L expression-mediated inhibition of migration and invasion abilities. Left, overexpression of IFI44L reduced the phosphorylation of Met, which could be partially rescued by transfecting Met vector. The actin was used as an internal control. Relative band intensity was quantified by ImageJ 1.42 and was represented with normalized mean ± s.e. (n = 3) below each band. Right, the migration and invasion abilities affected by overexpression of IFI44L and ectopic expression of Met in Hep3B cell line. Quantitative data are shown by histograms and representative photographs of the migrated/invaded cells from different treatments are shown. Histograms represent means ± s.d. from 3 independent experiments (*, P < 0.05; **, P < 0.01). (TIF 437 kb) [file 12885_2018_4529_MOESM6_ESM.tif]
